# Assessment of Posterolateral Lumbar Fusion: A Systematic Review of Imaging-Based Fusion Criteria

**DOI:** 10.2106/JBJS.RVW.22.00129

**Published:** 2022-10-26

**Authors:** A. Mechteld Lehr, Anneli A.A. Duits, Maarten R.L. Reijnders, Don Nutzinger, René M. Castelein, F. Cumhur Oner, Moyo C. Kruyt

**Affiliations:** 1 Department of Orthopaedic Surgery, University Medical Center Utrecht, Utrecht, The Netherlands

## Abstract

Noninvasive assessment of osseous fusion after spinal fusion surgery is essential for timely diagnosis of patients with symptomatic pseudarthrosis and for evaluation of the performance of spinal fusion procedures. There is, however, no consensus on the definition and assessment of successful posterolateral fusion (PLF) of the lumbar spine. This systematic review aimed to (1) summarize the criteria used for imaging-based fusion assessment after instrumented PLF and (2) evaluate their diagnostic accuracy and reliability.

**Methods:**

First, a search of the literature was conducted in November 2018 to identify reproducible criteria for imaging-based fusion assessment after primary instrumented PLF between T10 and S1 in adult patients, and to determine their frequency of use. A second search in July 2021 was directed at primary studies on the diagnostic accuracy (with surgical exploration as the reference) and/or reliability (interobserver and intraobserver agreement) of these criteria. Article selection and data extraction were performed by at least 2 reviewers independently. The methodological quality of validation studies was assessed with the QUADAS-2 (Quality Assessment of Diagnostic Accuracy Studies 2) and QAREL (Quality Appraisal of Reliability Studies).

**Results:**

Of the 187 articles included from the first search, 47% used a classification system and 63% used ≥1 descriptive criterion related to osseous bridging (104 articles), absence of motion (78 articles), and/or absence of static signs of nonunion (39 articles). A great variation in terminology, cutoff values, and assessed anatomical locations was observed. While the use of computed tomography (CT) increased over time, radiographs remained predominant. The second search yielded 11 articles with considerable variation in outcomes and quality concerns. Agreement between imaging-based assessment and surgical exploration with regard to demonstration of fusion ranged between 55% and 80%, while reliability ranged from poor to excellent.

**Conclusions:**

None of the available criteria for noninvasive assessment of fusion status after instrumented PLF were demonstrated to have both sufficient accuracy and reliability. Further elaboration and validation of a well-defined systematic CT-based assessment method that allows grading of the intertransverse and interfacet fusion mass at each side of each fusion level and includes signs of nonunion is recommended.

**Level of Evidence:**

Diagnostic Level III. See Instructions for Authors for a complete description of levels of evidence.

Spinal fusion surgery, with the aim of obtaining a solid fusion between vertebrae, is now an established treatment for numerous lumbar spine disorders, including mechanical instability, degenerative disease, and deformity. Over the past century, the surgical strategy has evolved from autologous bone grafting to use of rigid pedicle screw systems and/or interbody fusion devices in combination with bone graft substitutes^1-7^.

Accurate and reliable assessment of the postoperative fusion status is essential for timely diagnosis of patients with symptomatic pseudarthrosis who require additional treatment and for evaluation of the performance of spinal fusion procedures. Although multiple articles and systematic reviews have investigated the pros and cons of available imaging modalities to assess the fusion status after posterolateral fusion (PLF), and thin-slice computed tomography (CT) with multiplanar reconstructions is recommended, the criteria for successful fusion are still controversial. Moreover, evidence regarding the diagnostic accuracy of imaging-based fusion assessment methods is still limited. Numerous combinations of imaging modalities, criteria, and cutoff values to diagnose solid fusion are applied in clinical practice. This partially explains the wide range of fusion rates reported in the literature, and complicates the interpretation and comparison of different studies^8-13^. The aim of the current systematic review was to summarize and evaluate the criteria used for imaging-based fusion assessment after PLF of the lumbar spine.

## Materials and Methods

This systematic review is part of an investigation of both PLF and interbody fusion (IBF) of the lumbar spine and was performed with a 2-stage approach. The first stage aimed to identify reproducible criteria used for imaging-based fusion assessment and determine which are used most frequently in the literature. The second stage focused on the accuracy (with surgical exploration as reference) and reliability (in terms of interobserver and intraobserver agreement) of these criteria. This article solely reports on the results for PLF.

### Search Strategies

In the first stage, MEDLINE, Embase, and the Cochrane Central Register of Controlled Trials (CENTRAL) and Cochrane Database of Systematic Reviews (CDSR) were searched in November 2018 for literature published through that date. The second stage involved searches of MEDLINE and Embase in July 2021 for articles on accuracy and reliability. Key search terms are listed in Table I. The MEDLINE search string for each search, which was adapted for the searches of the other databases, can be found in Appendix A.

**TABLE I:** Key Search Term Categories*

Search 1: identification of reproducible criteria for imaging-based fusion assessment	
Population	Lumbar spine, degenerative (disc) disease, spondylolisthesis, spinal canal stenosis, spinal deformity
Intervention	Spinal fusion, posterolateral fusion, interbody fusion, bone grafts
Outcome	Fusion, nonunion
Search 2: accuracy and reliability of imaging-based fusion criteria	
Population	Lumbar spine, spinal fusion, posterolateral fusion, interbody fusion
Index test	Radiography, CT, DEXA, SPECT, PET, MRI
Reference test	Surgical exploration
Diagnosis	Fusion, nonunion
Outcome	Accuracy measures, reliability measures

* The full search strings are given in Appendix A. CT = computed tomography, DEXA = dual-energy x-ray absorptiometry, SPECT = single photon emission CT, PET = positron emission tomography, and MRI = magnetic resonance imaging.

### Article Selection

After removal of duplicates, the identified references were assessed for eligibility based on the title and abstract by 2 reviewers (A.A.A.D. and A.M.L.) independently using Rayyan^14^. The full text of potentially eligible articles was retrieved and checked for inclusion by the same reviewers using Zotero (version 5; Corporation for Digital Scholarship). Disagreements between the reviewers were resolved by discussion. If consensus could not be reached, a third reviewer (F.C.O.) was consulted.

In the first stage, primary human studies, including controlled trials, observational studies, and reports of multiple cases or case series, that described imaging-based criteria for fusion assessment after primary instrumented PLF between T10 and S1 in adult patients were considered. Studies were excluded if >50% of the patients met ≥1 of the following criteria: age of <18 years, cervical or main thoracic fusion, revision of instrumented spinal fusion, traumatic fractures, or pathological conditions such as tumor or infection. Studies were also excluded if the target population or method of fusion assessment was unclear, or if <10 patients met the inclusion criteria.

In the second stage, the inclusion criteria were primary human studies on the accuracy and/or reliability of reproducible imaging-based criteria for fusion assessment of the lumbar and/or thoracolumbar spine. Accuracy studies were considered only if the reference standard was surgical exploration. Studies about cervical fusion, comparing different imaging modalities, or with reliability as a secondary outcome measure were excluded. Studies that did not describe how spinal fusion was assessed were also excluded. Systematic reviews on the searched topic were only used to identify additional eligible articles cited as references.

### Data Extraction

In the first stage, the following data were extracted from the included articles using a predefined electronic form (Microsoft Excel, version 2016): year of publication, first and last author, imaging modality, description of and reference for the used fusion criteria, cutoff value for successful fusion, and whether accuracy and/or reliability were reported as secondary outcomes. Data extraction was divided among 4 reviewers (A.A.A.D., D.N., A.M.L., and M.R.L.R.).

In the second stage, data extraction (by A.A.A.D. and A.M.L.) also included the description of the study design and population, and (as far as applicable) the description of the surgical fusion assessment method, measures of accuracy (including percentage agreement between imaging-based assessment and surgical exploration, sensitivity [ability to detect true positives], specificity [ability to detect true negatives], positive predictive value [PPV], and negative predictive value [NPV]), and measures of reliability (interobserver and intraobserver agreement). All data extraction was checked by the other reviewer (A.A.A.D. or A.M.L.), and any discrepancies were discussed to reach consensus.

### Data Analysis

In both stages, interobserver reliability of the inclusion of full-text articles was assessed on the basis of the percentage agreement. Characteristics of the included articles were summarized using descriptive statistics. In stage 1, the frequency of use of each specific classification system or criterion was calculated on the basis of the absolute frequency (number of articles) and the corresponding relative frequency (percentage of all articles). Frequency analyses were also performed for the imaging modality that was used, use of combinations of criteria, and cutoff values for successful fusion. If articles in stage 2 reported raw data instead of percentage agreement, sensitivity, specificity, PPV, and/or NPV, these measures of accuracy were calculated to facilitate comparisons among the studies.

### Quality Assessment of Validation Studies

The methodological quality of the accuracy and reliability studies included in stage 2 was assessed using the QUADAS-2 (Quality Assessment of Diagnostic Accuracy Studies 2) and QAREL (Quality Appraisal of Reliability Studies) checklists, respectively^15,16^. When ≥1 signaling question of the QUADAS-2 was answered with “no” or “unclear,” the risk of bias of that domain was scored as “high” or “unclear,” respectively. The quality of reliability studies was considered high when ≥60% of the QAREL items were answered with “yes.”^17^

### Source of Funding

No financial support was received for this research.

## Results

### Stage 1: Identification of Criteria

#### Article Selection

**Fig. 1:**
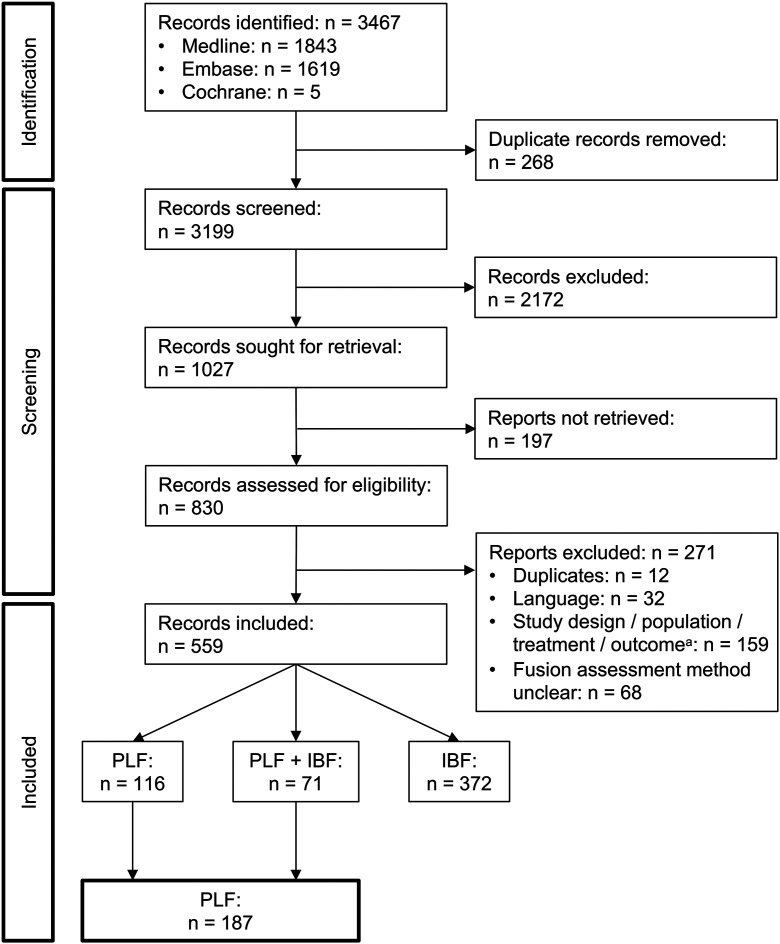
PRISMA (Preferred Reporting Items for Systematic Reviews and Meta-Analyses) flow diagram of identification, screening, and inclusion of articles in stage 1. PLF = posterolateral fusion, and IBF = interbody fusion. ^a^Hierarchical stratification of exclusion reasons: 36 articles, wrong study design; 40, wrong population; 55, wrong treatment; and 28, wrong outcome.

The first search yielded 3,199 unique articles (Fig. 1). A total of 830 full-text articles were assessed for eligibility and 559 articles were included, of which 187 involved PLF. It is noteworthy that 68 full-text articles (8%) were excluded because they did not describe how spinal fusion was assessed. Agreement between the reviewers was 89% based on the title and abstract and 85% based on the full text selection. Seventy-one (38%) of the included articles also discussed IBF (i.e., PLF in combination or comparison with IBF).

### Fusion Assessment Method

Study characteristics (including decade of publication, imaging modality used, and fusion assessment method) are summarized in Table II. Fusion was most commonly assessed using static radiographs (134 of the 187 articles; 72%), followed by dynamic radiographs (51%) and CT scans (35%). More than half (55%) of the articles used a combination of imaging modalities. The percentage of articles using CT increased from 14% in the 1990s to 48% in the 2010s.

**TABLE II:** Study Characteristics for the 187 Articles Included in Stage 1

	No. (%) of Articles
Decade of publication	
1990-1999	29 (16%)
2000-2009	71 (38%)
2010 -2018	87 (47%)
Imaging modality	
Static radiographs	134 (72%)
Dynamic radiographs	95 (51%)
Computed tomography	66 (35%)
Unclear	4 (2%)
Fusion assessment method	
Classification system	88 (47%)
Descriptive criteria	118 (63%)

Almost half (47%) of the articles used a classification or grading system, whereas 63% used ≥1 descriptive criterion such as osseous bridging and absence of signs of nonunion. Nineteen (10%) of the articles used both a classification and descriptive criteria.

### Classifications

Among the 88 articles that used a classification or grading system, the Lenke classification^18^ was reported most frequently (34%), followed by the Christensen classification^19^ (19%). The frequencies of other classifications are given in Table III. Examination of the references of the 22 articles that used a defined but unnamed grading system revealed that 4 articles used the grading system described by Singh et al.^20^ and 4 used the assessment method of Suk et al.^21^.

**TABLE III:** Classifications (Including Modified Versions) Identified in Stage 1*

Classification	No. (%) of Articles		
	Overall*	Stratified by Imaging Modality†	
		Radiographs	CT
Brantigan^49^	3 (3%)	1	2
Bridwell^22^	7 (8%)	6	1
Christensen^19^	17 (19%)	11	7
Glassman^23^	7 (8%)	1	7
Jorgenson^50^	4 (5%)	4	
Lenke^18^	30 (34%)	24	8
Miscellaneous	22 (25%)		
Singh^20^	4 (5%)	2	3
Suk^21^	4 (5%)	4	
Other‡	14 (16%)	11	3

* Absolute and relative frequency of reporting in the 88 articles that used a classification system for fusion assessment.

† Excluding 2 articles that were unclear about the imaging modality used. CT = computed tomography

‡ Classifications reported in a single article and therefore not further analyzed.

All of the classification systems evaluate continuity of osseous bridging on a 3, 4 or 5-point scale. Interestingly, the quality of bridging is named in a variety of ways, without clearly citing a previous article or other source on which the terminology was based, including *trabecular, mature, dense, solid, amorphous,* and *cortical edges*. Some classifications make a distinction between unilateral and bilateral PLF^18,22,23^. The method by Singh et al.^20^ also includes absence of signs of nonunion as a criterion for successful fusion, whereas Suk et al.^21^ combine osseous bridging with <4° of intersegmental motion. The grades that were considered to indicate successful fusion (e.g., Lenke A [bilateral solid fusion mass] or Lenke A and B [bilateral and unilateral solid fusion mass]) also varied widely among studies, as did whether only intertransverse fusion or also facet fusion was assessed.

### Descriptive Criteria

A variety of descriptive criteria for fusion assessment were extracted from 118 articles (see Appendix B). These fell into 3 basic categories: (1) continuity of osseous bridging, (2) absence of motion, and (3) absence of static signs of nonunion (Table IV). Criteria related to continuity of osseous bridging were most frequently used (88%), followed by absence of motion (66%) and absence of static signs of nonunion such as radiolucency around the screws (33%). Twenty-nine (25%) of the articles solely considered criteria involving continuity of osseous bridging. Combinations of criteria were used in 78 articles (Table V).

**TABLE IV:** Categorical Descriptive Criteria Identified in Stage 1

Categorical Criteria	No. (%) of Articles		
	Overall	Stratified by Imaging Modality†	
		Radiographs	CT
Continuity of osseous bridging	104 (88%)	80	37
Absence of motion	78 (66%)	78	
Absence of static signs of nonunion	39 (33%)	31	11
Miscellaneous	3 (3%)	3	1

* Absolute and relative frequency of reporting in the 118 articles that used descriptive criteria for fusion assessment. †Excluding 2 articles that were unclear about the imaging modality used. CT = computed tomography.

**TABLE V:** Combinations of Descriptive Criteria

Continuity of Osseous Bridging	Absence of Motion	Absence of Static Signs of Nonunion	No. of Articles
Yes	Yes	Yes	25
Yes	Yes	No	39
Yes	No	Yes	9
No	Yes	Yes	5

Further exploration of the 104 articles that included continuity of osseous bridging revealed that 50% particularly assessed osseous bridging between the transverse processes and only 9% assessed facet fusion (see Appendix B). As with the classifications described above, the terminology regarding the quality of osseous bridging and the definition of successful fusion (unilateral or bilateral) varied widely.

Reported cutoff values for the absence of rotational motion (1.5° to 10°) and translational motion (2 to 4.5 mm) on dynamic radiographs (flexion-extension) varied considerably (see Appendix B). In addition, the exact method for measuring intersegmental motion was often not described. Static signs of nonunion were defined as implant failure or loosening, radiolucency around the implant, and a gap, cleft, or line within the fusion mass on imaging.

### Combination of Classification and Descriptive Criteria

Of the 19 articles that used both a classification and descriptive criteria, 26% reported separate fusion rates based on the different assessment methods. The majority (68%) used continuity of osseous bridging (assessed with a classification system on CT scans or radiographs) and absence of motion (assessed using dynamic radiographs) as combined criteria for successful fusion. Three of these articles used absence of implant failure as a third criterion.

### Stage 2: Accuracy and Reliability

#### Article Selection

**Fig. 2:**
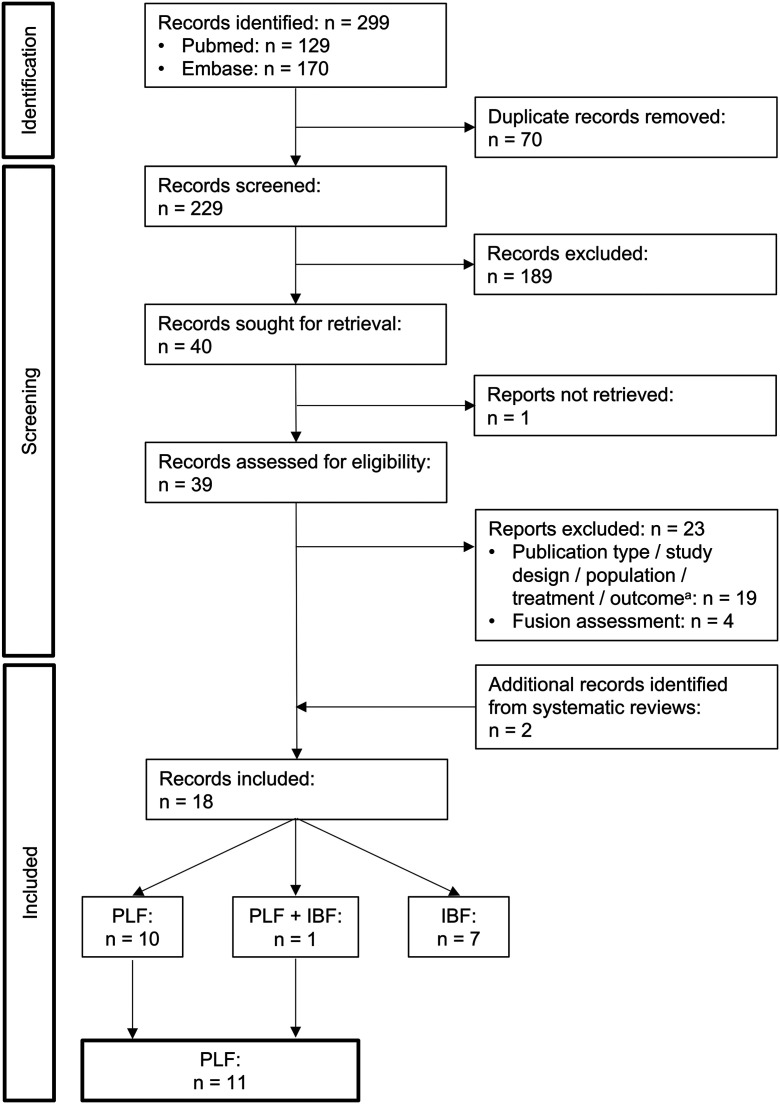
PRISMA flow diagram of identification, screening, and inclusion of articles in stage 2. PLF = posterolateral fusion, and IBF = interbody fusion. ^a^Hierarchical stratification of exclusion reasons: 9 articles, wrong publication type (abstract or review); 7, wrong study design; 1, wrong treatment; and 2, wrong outcome.

The flow diagram of the second search is shown in Figure 2. Agreement between the 2 reviewers was 82% for selection of the 229 articles based on the title and abstract and 90% for selection of the 39 articles based on the full text. Checking the reference list of the 6 systematic reviews that were identified by this search yielded 2 additional included articles (both on IBF). A total of 18 articles were included, of which 11 reported on PLF. The study design, method of fusion assessment, and measures of accuracy and/or reliability for these 11 articles are summarized in Table VI.

**TABLE VI:** Studies Investigating the Diagnostic Accuracy and/or Reliability of Imaging-Based Assessments of Posterolateral Fusion*

Study	Study Design	Fusion Assessment	Fusion Outcome	Accuracy or Reliability Measures
Accuracy studies				
Kant (1995)^26^	Retrospective cohort study of 75 patients (126 levels) who had unintentional instrumentation removal (mean, 51 wk; range, 1 mo-212 wk) after instrumented lumbar PLF.	Imaging-based fusion: solid bone between transverse processes or obliteration and fusion of the facet joint on oblique views on static radiographs. Surgical exploration: no motion (as determined by 4 different methods) and visualization of a solid bone mass from the ala to the transverse process, or total obliteration of the facet joint.	Imaging-based fusion rate: 78%. Surgical exploration: 87 levels (69%) were fused.	Agreement between imaging and surgical exploration: poor (70.6%) overall agreement (mean kappa = 0.26; 95% CI, 0.07-0.44; range, 0.03-0.59). Accuracy for detecting fusion: sensitivity = 85%, specificity = 38%, PPV = 76%, NPV = 54.
Larsen (1996)^28^	Prospective cohort study of 25 symptomatic patients (41 levels) who underwent hardware removal >1 yr after instrumented lumbar PLF.	Imaging-based fusion: presence of bridging bony trabeculae on static radiographs (anteroposterior, lateral, and oblique views; 21 patients), <3° of motion on dynamic radiographs (11 patients), presence of bridging bony trabeculae on thick-slice (5-mm) CT (sagittal and coronal reformations; 24 patients), lack of increased uptake on bone scintigraphy (20 patients). Surgical exploration: inspection of the fusion mass for solid fusion or pseudarthrosis.	Imaging-based fusion rates: 29% (static radiographs), 91% (dynamic radiographs), 42% (CT), 80% (bone scintigraphy). Surgical exploration: 16 patients were classified as having fusion (64%), 9 patients were diagnosed with pseudarthrosis.	Agreement between imaging and surgical exploration: 62% (static radiographs), 55% (dynamic radiographs), 63% (CT), 60% (bone scintigraphy). Accuracy for detecting fusion: sensitivity = 42% (static radiographs), 86% (dynamic radiographs), 53% (CT), 83% (scintigraphy) for detecting fusion. Specificity = 89% (static radiographs), 0% (dynamic radiographs), 78% (CT), 25% (scintigraphy). PPV = 83% (static radiographs), 60% (dynamic radiographs), 80% (CT), 63% (scintigraphy). NPV = 53% (static radiographs), 0% (dynamic radiographs), 50% (CT), 50% (scintigraphy).
Jacobson (1997)^25^	Prospective cohort study of 10 symptomatic patients (20 sites) who underwent second-look surgery ≥9 mo after (thoraco)lumbar PLF.	Fusion on ultrasound: solid fusion was indicated by the presence of an echogenic and shadowing interface that bridged the transverse processes, facets, laminae, or spinous processes. Surgical exploration: visual presence of bridging bone. In case of a cleft or indentation, pressure was applied to assess for motion.	Fusion on ultrasound: solid fusion was depicted at 6 of 20 sites. Surgical exploration: 10 sites were considered fused.	Agreement between imaging and surgical exploration: 80%. Accuracy for detecting pseudarthrosis: sensitivity = 100%, specificity = 60%, PPV = 71%, NPV = 100%.
Kanayama (2006)^29^	RCT of 19 patients with single-level instrumented lumbar PLF and per-protocol instrumentation removal when the radiographic fusion criteria were met.	Imaging-based fusion: <5° of angular motion and <2 mm of translation at the operative level on dynamic radiographs, and evidence of posterolateral bridging bone on CT. Surgical exploration: intraoperative manipulation after instrumentation removal.	Imaging-based fusion rate: 84%. Surgical exploration: 11 (69%) of the 16 patients with radiographic fusion were considered to have a solid fusion.	Agreement between imaging and surgical exploration: 69% overall agreement.
Accuracy and reliability studies				
Carreon (2007)^27^	Retrospective cohort study of 93 patients (163 levels) who had revision surgery (mean, 49 ± 38 mo; range, 1-148 mo) after instrumented lumbar PLF.	Imaging-based fusion: obliteration of the facet joint space (facet fusion) and continuous trabeculated bone connecting the transverse processes (posterolateral gutter fusion) on fine-cut (1-mm) CT (sagittal and coronal reconstructions). Assessment by 3 observers. Surgical exploration: absence of bony continuity on inspection of the posterolateral gutters and facets and the presence of motion under distraction were defined as a nonunion.	Imaging-based fusion: facet fusion was bilateral in 71% and unilateral in 8%, posterolateral gutter fusion was bilateral in 43% and unilateral in 14%. Surgical exploration: the rate of nonunion was 20%.	Accuracy: likelihood ratio for solid fusion at surgical exploration was 2.90 when radiographic facet fusion was bilateral, 0.55 when facet fusion was unilateral, 0.19 when both facets were not fused, 8.31 when posterolateral gutter fusion was bilateral, 5.37 when posterolateral fusion was unilateral, and 0.35 when both posterolateral gutters were not fused. PPV for solid fusion = 74% for bilateral facet fusion, 35% for unilateral facet fusion, 16% for no facet fusion, 89% for bilateral posterolateral gutter fusion, 84% for unilateral posterolateral gutter fusion, 26% for no posterolateral gutter fusion. Interobserver reliability: moderate for facet fusion (kappa = 0.42) and substantial for posterolateral gutter fusion (kappa = 0.62).
Fogel (2008)^24^	Retrospective cohort study of 90 patients (172 levels) who had unplanned surgical exploration (mean, 27 mo; range, 12-65 mo) after combined lumbar IBF and instrumented PLF.	Imaging-based fusion: Lenke classification of Grade A on static radiographs (anteroposterior and lateral views; 2 observers) and thin-section (1-mm) helical CT (sagittal and coronal reconstructions). Surgical exploration: observation of a solid cortical posterolateral bone bridge and absence of visible motion.	Imaging-based fusion rates: 75% (static radiographs), 68% (CT). Surgical exploration: 97% fusion rate.	Agreement between imaging and surgical exploration: 77% (static radiographs) and 72% (CT). Accuracy for detecting pseudarthrosis: sensitivity = 100% (static radiographs and CT). Specificity = 77% (static radiographs), 70% (CT). PPV = 9% (static radiographs), 11% (CT). NPV = 100% (static radiographs and CT). Interobserver reliability: overall, 99% for IBF and PLF on static radiographs.
Spirig (2019)^31^	Prospective cohort study of 41 patients (159 pedicle screws) who underwent revision surgery (mean, 3.0 ± 3.1 yr; range, 0-13.5 yr) after instrumented lumbar PLF.	Imaging-based pseudarthrosis: presence of peri-screw edema on MRI (2 observers) and presence of peri-screw osteolysis on static radiographs (anteroposterior and lateral views) and thin-slice (2-mm) CT (2 observers). Surgical exploration: screw loosening was defined as an unscrewing torque of ≤60 N-cm.	Imaging-based pseudarthrosis: screw loosening rates ranged between 31% and 36%. Surgical exploration: 35% of the screws were loose.	Accuracy for screw loosening: sensitivity = 34.5%-43.9% (MRI), 54.2% (static radiographs), 52.4%-64.8% (CT). Specificity = 77.4%-92.1% (MRI), 83.5% (static radiographs), 93.8%-96.7% (CT). Interobserver reliability: poor for MRI (kappa = 0.289. Intraclass correlation analysis showed good agreement for CT (ICC = 0.860).
Reliability studies				
Christensen (2001)^19^	RCT of 43 patients (53 levels) with instrumented versus 36 patients (50 levels) with noninstrumented lumbar PLF (1 yr follow-up).	Imaging-based fusion: Christensen classification (unilateral or bilateral fusion at all intended levels) on static radiographs (anteroposterior and lateral views). Assessment by 4 observers and repetition after 8 wk.	Imaging-based fusion: mean fusion rate was 81% (70%-82% for instrumented fusion, and 81%-92% for noninstrumented fusion). 68% of the patients were classified as having fusion at all levels by all 4 observers.	Interobserver reliability: mean agreement = 87% (range, 83%-93%), mean kappa = 0.58 (range, 0.44-0.70; fair to good). Intraobserver reliability: mean agreement = 93% (mean kappa = 0.76; excellent).
Tokuhashi (2008)^32^	Retrospective cohort study of 190 patients with instrumented lumbar PLF with or without additional IBF (mean follow-up, 5.7 yr; range, 3-13 yr).	Imaging-based pseudarthrosis: clear zone (≥1-mm circumferential radiolucency around a screw confirmed in ≥2 directions by >2 of 3 observers) on static radiographs. Assessment was repeated after 4 wk.	Imaging-based pseudarthrosis: The clear-zone-positive rate decreased from 41% (78 patients) at 6-mo follow-up to 15% (28 patients) at ≥3-yr follow-up.	Interobserver reliability: mean agreement = 96.2% (range, 95.1%-96.8%), mean kappa = 0.90 (range, 0.87-0.91; excellent). Intraobserver reliability: mean agreement = 97.4% (range, 96.8%-97.9%), mean kappa = 0.95 (range, 0.93-0.96; excellent).
Dakhil-Jerew (2009)^33^	Prospective cohort study of 50 patients with dynamic posterolateral pedicle screw stabilization (260 screws) (mean follow-up, 40.9 mo; range, 20-74 mo).	Screw loosening: assessed on static radiographs (anteroposterior and lateral) based on a “halo zone sign” (7 observers) and a “double-halo sign” (4 observers).	Screw loosening: 3%-44% based on “halo zone sign” and 7%-9% based on “double-halo sign.”	Interobserver reliability: poor agreement for “halo zone sign” (kappa = 0.1462 [95% CI, 0.0332-0.2592]) and substantial agreement for “double-halo sign” (kappa = 0.666 [95% CI, 0.496-0.836]).
Gotfryd (2014)^30^	Prospective cross-sectional study of 20 patients with instrumented lumbar PLF (minimum follow-up, 24 mo; mean, 32 mo).	Imaging-based fusion: Christensen classification (unilateral or bilateral fusion) on static radiographs (anteroposterior and lateral) and <5° difference in Cobb angle on dynamic radiographs. Assessment by 6 observers; 4 observers repeated the assessment after 8 wk.	Not reported	Interobserver reliability: based on the first rating, mean agreement = 76% ± 7.8% (kappa = 0.07-0.50; poor to moderate) for static radiographs and 78% ± 9.1% (kappa = −0.08-0.50; poor to moderate) for dynamic radiographs. Intraobserver reliability: mean agreement = 63% ± 10% (kappa = 0.06-0.26; poor to reasonable) for static radiographs and 84% ± 10% (kappa = 0.20-0.73; poor to substantial) for dynamic radiographs.

* Accuracy studies are listed first, then reliability studies. Within each category, studies are listed in order of publication year. PLF = posterolateral fusion, CT = computed tomography, CI = confidence interval, PPV = positive predictive value, NPV = negative predictive value, RCT = randomized controlled trial, IBF = interbody fusion, MRI = magnetic resonance imaging, and ICC = intraclass correlation coefficient.

### Diagnostic Accuracy

Of the classifications identified in stage 1, only the Lenke classification was assessed for diagnostic accuracy, by 1 retrospective study^24^. With bilateral fusion masses (Lenke A) considered to indicate successful PLF, the fusion rate determined with surgical exploration (97%) was underestimated by both radiography (75%) and CT (68%). Agreement with surgical exploration was 77% for radiography and 72% for CT. The sensitivity for detecting pseudarthrosis was 100% for both imaging modalities; specificity was 77% for radiographs and 70% for CT scans^24^.

Five studies evaluated continuity of osseous bridging, and 2 of them also included absence of motion as a criterion for successful fusion^25-29^. The single study that used ultrasound to evaluate PLF (between the transverse processes, facets, laminae, or spinous processes) reported 80% agreement between ultrasound and surgical exploration for clinically suspected pseudarthrosis; the sensitivity and specificity of ultrasound for detecting nonunion were 100% and 60%, respectively^25^. Kant et al. considered solid bone from a transverse process to the adjacent transverse process or obliteration and fusion of the facet joint on radiographs as solid fusion. Overall agreement between radiographs and surgical exploration after instrumentation removal was poor, with a mean kappa of 0.26 (95% confidence interval [CI], 0.07 to 0.44; range, 0.03 to 0.59). Sensitivity for detecting solid fusion was 85%, and specificity was 38%^26^. Carreon et al. investigated the accuracy of facet fusion (obliteration of the facet joint space) and posterolateral gutter fusion (continuous trabeculated bone connecting the transverse processes) using fine-cut CT scans in patients who underwent revision surgery. The likelihood ratio was higher for bilateral facet fusion (2.90) than for unilateral fusion (0.55). For posterolateral gutter fusion, these likelihood ratios were better (8.31 and 5.37, respectively)^27^.

Larsen et al. compared the presence of bridging osseous trabeculae on static radiographs and thick-slice CT scans with operative findings after instrumentation removal. In addition, they reported on the diagnostic accuracy of dynamic radiographs (<3° of motion) and bone scintigraphy (lack of increased uptake). Depending on the imaging modality, the radiographic fusion rate ranged between 29% (static radiographs) and 91% (dynamic radiographs), while the surgically determined fusion rate was 64%. Bridging osseous trabeculae on static radiographs had the lowest sensitivity (42%) but the highest specificity (89%) for detecting fusion. In contrast, dynamic radiographs had the highest sensitivity (86%) but 0% specificity. Agreement with surgical exploration was highest for CT (63%)^28^. Kanayama et al. evaluated posterolateral bridging bone on CT in combination with <5° of angular motion and <2 mm of translation on dynamic radiographs. In that randomized controlled trial, instrumentation was removed if the radiographic fusion criteria were met at the 1-year follow-up. However, the surgically determined fusion rate among these patients was only 69%^29^.

Spirig et al. evaluated the accuracy of pedicle screw loosening on radiographs, CT scans, and magnetic resonance imaging (MRI) by intraoperative measurement of unscrewing torque using a torque meter. The sensitivity for detecting screw loosening was 54% for radiographs, 52% to 65% for CT, and 35% to 44% for MRI. The specificity of these imaging modalities was 84%, 94% to 97%, and 77% to 92%, respectively^31^.

### Interobserver and Intraobserver Reliability

Two studies investigated the reliability of the Christensen classification on static radiographs^19,30^. Christensen et al. reported fair to good agreement among 3 observers (mean, 87% [range, 83% to 93%], kappa = 0.58 [range, 0.44 to 0.70]) and excellent intraobserver agreement based on an 8-week interval (mean, 93%; kappa = 0.76)^19^. Gotfryd et al. included 6 observers with different levels of expertise, with a mean interobserver agreement of 76% ± 7.8% (kappa = 0.07 to 0.50). Based on 4 observers and an interval of 8 weeks, mean intraobserver agreement was 63% ± 10% (kappa = 0.06 to 0.26). That study also reported on the interobserver agreement (78% ± 9.1% [kappa = −0.08 to 0.50]) and intraobserver agreement (84% ± 10% [kappa = 0.2 to 0.73]) for the fusion criterion of <5° difference in the Cobb angle on dynamic radiographs^30^.

The previously described CT-based accuracy study by Carreon et al. reported moderate agreement among 3 observers for facet fusion (kappa = 0.42) and substantial agreement for posterolateral gutter fusion (kappa = 0.62)^27^. The 3 studies that investigated radiolucency or osteolysis around the pedicle screws as a sign of pseudarthrosis showed poor to excellent interobserver or intraobserver reliability (Table VI)^31-33^.

Reliability measures were also reported as a secondary outcome in 10 of the articles from stage 1, and they ranged from good to excellent (see Appendix C).

### Quality Assessment of Validation Studies

The results of the QUADAS-2 and QAREL checklists are shown in Appendix D. All diagnostic accuracy studies were at risk for bias, mainly due to insufficient information about the selection of patients and poor description or variable application of the surgical exploration technique. Moreover, 3 studies had an inappropriate time interval between the diagnostic imaging and surgical exploration^27,29,31^.

The quality of all but 1 of the reliability studies was rated as high^32^. However, most articles did not explicitly mention whether raters were blinded to the findings of other raters (item 3), their own prior findings (item 4), and/or clinical information (item 6), as well as whether the order of images was varied in cases of repeated assessment (item 8). Three articles did not report a measure of precision for the estimate of reliability^24,27,31^.

## Discussion

Reliable assessment of the status of osseous fusion after spinal fusion surgery is imperative, as this is the intended outcome. Although open surgical exploration is considered the gold standard, clinicians and researchers must rely on noninvasive diagnostic imaging for routine assessment. There is, however, no consensus on the definition and assessment of successful fusion, and available imaging techniques all have their own advantages and limitations. As a consequence, reported fusion rates are based on a variety of criteria and cutoff values, often with limited clinical evidence. In an attempt to improve the determination of successful PLF of the lumbar spine, we systematically reviewed the imaging-based criteria employed in previous studies as well as their diagnostic accuracy and reliability.

Not surprisingly, the presence of a continuous osseous bridge between adjacent vertebrae was the most common criterion for successful fusion, but the terminology for the quality of osseous bridging and the assessed anatomical locations varied greatly. Although this review focused on rigidly instrumented fusions, many of the studies (51%) assessed angular or translational motion using dynamic radiographs, a method that has been shown to be inaccurate^28,34^. Recognized pitfalls of this method include the lack of normative data, use of a wide range of cutoff values, and measurement error (during both image capture and analysis)^35-38^. Moreover, absence of any intervertebral movement does not necessarily correspond with solid fusion. Interestingly, the upper limits of <3 mm of translational and <5° of angular motion are accepted for successful lumbar fusion by the U.S. Food and Drug Administration guidance for the evaluation of spinal systems, without citation of any reference^39^. The frequent use of the Lenke classification and Christensen classification (for both radiographs and CT scans) is supported by their good to excellent reliability for experienced observers, although evidence of diagnostic accuracy is very limited. While both classifications evaluate the presence of a continuous osseous bridge, the Lenke classification also includes the quality of the fusion mass and distinguishes between unilateral and bilateral fusion.

While our systematic review showed that the use of CT increased over time, static radiographs were the predominant imaging modality. Although CT is suggested to be better than radiographs, superiority of this imaging modality could not be demonstrated. A wide range of sensitivity and specificity values (for detecting fusion or nonunion) was observed, and agreement between imaging-based assessment and surgical exploration for identifying fusion was generally <80%^10-12,24,26,28,31,34,40,41^. This might be explained by the publication dates and retrospective design (i.e., use of data from decades ago) of most of the included accuracy studies. We expect this to change because image quality has improved greatly with the rapid advancement of CT techniques, such as helical scanning, higher resolution, multiplanar reconstructions, and reduced artifacts from implants.

One of the challenges in the search for the optimal assessment method involves the investigation of diagnostic accuracy. Since rigid instrumentation is typically not removed, surgical exploration of the fusion mass is often limited to symptomatic patients who qualify for revision surgery. This implies selection bias. Nevertheless, it would be interesting to apply different classifications or criteria to the same set of images and compare the outcomes with those of surgical exploration to determine which assessment method is most appropriate^27^. So far, such studies have mainly focused on the comparison of different imaging modalities^10^. It is also noteworthy that 4 frequently cited studies on the diagnostic accuracy of imaging-based fusion assessment could not be included in the current review as they did not describe criteria for successful fusion^34,40-42^.

In spite of the extensiveness of this systematic review, some limitations should be noted. The full text of many non-English-language articles could not be retrieved, resulting in the inclusion of only English-language articles. For the same reason, articles published before 1990 were not included. Given the large number of articles, data extraction from the articles included in stage 1 was limited to the fusion assessment method; data about the study design, sample size, and population were not considered.

### Recommendations

Although its superiority in fusion assessment could not be demonstrated by this systematic review, thin-slice CT with multiplanar reconstructions is considered the most appropriate modality when symptomatic pseudarthrosis is suspected and when performing clinical studies with radiographic fusion as the primary outcome. In our opinion, dynamic radiographs have no added value in the presence of rigid instrumentation. Classification systems such as those of Lenke and Christensen showed good reliability for the systematic assessment of posterolateral osseous bridging, but the terminology that is widely used for osseous bridging can be subjective. Moreover, discrimination between intertransverse and facet fusions, and whether unilateral or bilateral fusion is considered successful, have been shown to be relevant. Therefore, we propose the use of a systematic approach that specifies the particular anatomical locations to be assessed in multiple planes and allows grading of the quality of the fusion mass at each side of each fusion level. Whether this should be done in perpendicular or reconstructed planes and what terminology is most appropriate remain to be studied. We also recommend including signs of nonunion in the classification, as their presence precludes solid fusion. The findings from this systematic review were, however, not conclusive regarding which signs and assessment method are most predictive.

### Further Research

This systematic review showed that none of the available criteria for noninvasive assessment of fusion after instrumented PLF have been demonstrated to have both sufficient accuracy and reliability. Further elaboration of a well-defined and detailed systematic assessment method is a prerequisite for both clinical practice and research. Recognizing the limited feasibility of surgical exploration as the reference standard, further research should be directed at studying the accuracy of assessment methods using the current generations of CT scanners and implants, as well as the location of imaging-based fusion (between transverse processes or between facets) that correlates best with a true solid fusion^27,43,44^. Another underexplored aspect, related to the quality of the fusion mass, is the distinction between (potential) ongoing bone formation and nonunion. Given the tremendous technological advancements in the automated classification of degenerative discs, spinal deformities, and spinal fractures, we also expect the assessment of spinal fusion to profit from artificial intelligence^45-47^. In addition to CT, the applicability of radiation-free imaging modalities such as MRI and ultrasound should continue to be explored^25,48^.

## Appendix

Supporting material provided by the authors is posted with the online version of this article as a data supplement at jbjs.org (http://links.lww.com/JBJSREV/A872).
